# The relationship between workplace bullying and professional self‐concept in Iranian nurses

**DOI:** 10.1002/nop2.622

**Published:** 2020-09-17

**Authors:** Amin Hosseini, Seyed Hossein Mousavi, Fatemeh Hajibabaee, Shima Haghani

**Affiliations:** ^1^ Medical‐Surgical Nursing School of Nursing and Midwifery Tehran University of Medical Sciences Tehran Iran; ^2^ Psychiatric Nursing School of Nursing and Midwifery Tehran University of Medical Sciences Tehran Iran; ^3^ School of Nursing and Midwifery Tehran University of Medical Sciences Tehran Iran; ^4^ Biostatistics Nursing Care Research Center Iran University of Medical Sciences Tehran Iran

**Keywords:** Iran, nursing, professional self‐concept, workplace bullying

## Abstract

**Aim:**

To determine the level of workplace bullying among Iranian nurses and its relationship with their professional self‐concept.

**Design:**

This was a descriptive correlational study.

**Methods:**

This study was performed on 200 nurses working in the emergency departments and intensive care units of Imam Khomeini Hospital Complex affiliated to Tehran University of Medical Sciences, Iran, in 2020. The participants were chosen using the simple random sampling method and data collection tools were Negative Acts Questionnaire—Revised and Nurse Self‐Concept Questionnaire. The Pearson correlation coefficient was used to determine the relationship between workplace bullying and professional self‐concept.

**Results:**

Based on the experience of daily or weekly workplace bullying, the mean scores of workplace bullying in nurses in terms of work‐related bullying, person‐related dimension and physical intimidation were 10.11%, 4.27% and 5.66%, respectively, and the overall mean score was 6.68%. The results of this study also showed that workplace bullying is inversely related to professional self‐concept and almost all of its dimensions (*p* = .002, *r* = −.219).

## 1 INTRODUCTION

The working environment of healthcare providers and hospitals is always subject to high work pressure, complexity and chaos. It is constantly changing due to developments in various fields of treatment and care (Parchment & Andrews, 2019). Meanwhile, the phenomenon of bullying is a serious problem in these organizations. Workplace bullying is a combination of tactics where a wide range of behaviours and communications are used. These repeated behaviours are detrimental to health and occur in the form of verbal insults, threats, insults, humiliation, neglect, intimidation, sabotage and a combination of these that disrupt the flow of work in the workplace (Hogh, Baernholdt, & Clausen, 2018). Bullying is the abusive behaviour that an individual or a group of people repeatedly commit. Generally, workplace bullying occurs when a person has experienced at least two negative behaviours in one week (Johnson, 2009). The term "bullying" has been used in words such as violence, indecency, argument, harassment, harassment and aggression in different countries (Difazio et al., 2019; Wright & Khatri, 2015a, 2015b); however, the concept of "violence" is different from bullying, because violence is a behaviour that aims to inflict damage on one or more phenomena (Parchment & Andrews, 2019).

Workplace bullying in the nursing profession can cause symptoms such as irritability, depression, anxiety, fatigue, decreased concentration and self‐esteem and increased use of psychotropic substances and suicide and post‐traumatic stress disorder (Fang, Hsiao, Fang, & Chen, 2020; Karatza, Zyga, Tziaferi, & Prezerakos, 2016, 2017). It also creates huge costs for organizations, including increasing financial costs. Research shows that stress caused by workplace bullying has serious financial consequences. One of the important effects of workplace bullying in nursing is to create negative perceptions about yourself and your career. This phenomenon leads to negative emotions and thought by causing occupational burnout, depression and mental disorders, and in fact, this can affect nurses' professional self‐concept (Lee & Kim, 2018; Lu et al., 2019; Murray, 2009).

## 2 BACKGROUND

Numerous studies have shown that health organizations are among the work environments where high levels of organizational bullying are reported. This phenomenon has also been reported around the world as an important problem in the nursing profession (Wolf, Perhats, Clark, Moon, & Zavotsky, 2018). Oppressive behaviours in the nursing profession towards the nurse can be manifested by a physician or manager to a nurse, a nurse to a colleague, a nurse to a patient and a patient to a nurse. Statistics for this phenomenon have been reported in different countries for reasons such as conceptual differences, study design, cultural issues, public or private healthcare organizations and attitudes towards this phenomenon (Difazio et al., 2019). For example, bullying can be more common in some cultures. In some studies, physical bullying has been reported more frequently among Korean nurses. However, some previous studies have reported the prevalence of this dimension of bullying in Western countries to a small extent (Oh, Uhm, & Yoon, 2016). In addition, organizational culture is also effective in the prevalence of bullying. In some healthcare organizations, bullying behaviours are ignored by nursing managers and nursing staff may find these behaviours appropriate, because bullying can be a learned behaviour. Thus, it is important to consider the cultural differences in different countries and organizations in examining the prevalence of bullying (Whitney Wright & Naresh Khatri, 2015). A study in Turkey found that 21% of nurses experienced workplace bullying (Yıldırım, 2009). Another study in Turkey found that nurses in intensive care units were more vulnerable to bullying than other nurses (Efe & Ayaz, 2010). In the United States, 27.3% of emergency nurses have experienced workplace bullying. Other studies in the United States have reported 80 per cent of bullying (Lucena et al., 2018; Wolf et al., 2018). In Iran, a study found that 9% of nurses frequently experience workplace bullying (Esfahani & Shahbazi, 2014). Some researchers indicated that high prevalence of bullying in clinical settings that require technical expertise and stressful areas such as ICU (Vessey, DeMarco, & DiFazio, 2011).

Self‐concept can be considered the mental image of a person of himself and includes the individual's assessments of his progress, others' evaluations and social comparisons regarding success or failure. Professional self‐concept has been proposed in connection with work, and its evaluation in the organizational environment is highly necessary as it helps with individuals' success in organizations.

Nurses’ self‐concept is the information and beliefs that they have regarding themselves, their values and behaviours and is an attitude that can promotes the professional identity and create professionalism (Chang & Yeh, 2016), as nurses with a negative professional self‐concept describe the nursing profession as a sad and unsatisfactory profession and underestimate their qualification. If a person's perception of their profession is positive, they will be more psychologically prepared to accept professional responsibility, thus improving their job quality (Cao, Chen, Tian, & Diao, 2016). Nurses' self‐concept is one of the types of professional self‐concept, not only does it reflect the nurses' understanding of their professional competencies, but it also describes the practical skills necessary to care for patients (Losa‐Iglesias, Lopez Lopez, Rodriguez Vazquez, & Becerro de Bengoa‐Vallejo, 2017). Some factors such as age, work experience, university degree, job position and managerial background of the nurse and the work environment and related events such as communication and interaction with colleagues, superiors, colleagues in other health professions such as physician, relationship with the patients and their family and workload are the most important factors affecting the self‐concept of the nursing profession. In this regard, the presence of bullying in the workplace can have a devastating effect on the level of self‐concept of nurses (Lee & Kim, 2018; Shan et al., 2015). Since workplace bullying is a global problem in the nursing profession, numerous studies have been conducted in this regard in various countries around the world, including Canada, the United States, Australia, New Zealand, Pakistan and Turkey (Johnson, 2009). However, in Iran, most studies on bullying have been conducted on students and teachers in schools and few studies have been conducted among nurses. Given that there are no accurate statistics on the rate of workplace bullying among Iranian nurses and considering that professional self‐concept is one of the important issues that can be affected by the phenomenon of bullying, so this study aims to determine the amount of workplace bullying experienced by Iranian nurses and its connection with their professional self‐concept.

## 3 METHODS

### 3.1 Design

This study was a cross‐sectional descriptive correlational study conducted in 2020 with the aim of determining the rate of workplace bullying experienced by Iranian nurses and its relationship with professional self‐concept.

### 3.2 Sample and setting

The study was performed among 200 nurses working in the emergency department and intensive care units of Imam Khomeini Hospital Complex affiliated to Tehran University of Medical Sciences, Iran, who were selected by simple random sampling. Since this centre is one of the most comprehensive educational, research and medical centres in Iran, it was selected as the research centre. The hospital complex includes three hospitals. Some evidence reported that bullying may be more common in intensive care units (Efe & Ayaz, 2010; Vessey et al., 2011; Vessey, DeMarco, Gaffney, & Budin, 2009). Other studies have also indicated a high prevalence of bullying in hospital emergency departments due to the high volume and workload of nurses (Al‐Ghabeesh & Qattom, 2019). For these reasons, these wards were selected as the research environment. To determine the sample size at 95% confidence level and 80% test power and considering correlation coefficient between nurses' workplace bullying and professional self‐concept (*r* = .2) (In order for the relationship between variables to be considered statistically significant), after quantification in the formula, the sample size was estimated (*n* = 200). The inclusion criteria included employment as a nurse in one of the emergency departments and the intensive care unit of Imam Khomeini Hospital Complex, willingness to participate in the study and having at least 6 months of work experience as a nurse in that department. The exclusion criteria consisted of unwillingness to participate in the study and incomplete completion of the questionnaires. In this study, based on the list of nurses working in the emergency and intensive care units in the nursing office, several nurses who had the inclusion criteria to participate in the research were randomly selected for this purpose. The lottery method was used, such that each nurse was assigned a number and the numbers were written on a piece of paper and placed inside a box; then, the papers were taken out one by one until the desired sample size was completed. It should be noted that the number of samples in each ward was proportional to the total number of nurses in that ward who had inclusion criteria.

### 3.3 Data collection

Data collection tools included a demographic information form (items on age, gender, marital status, how entered the field of nursing, level of education, length of service, type of employment, working shift, duration of employment in the current department, management position, interest in the working department and existence of coercion for working in the department), the Negative Acts Questionnaire—Revised (NAQ‐R) and the Nurse self‐concept questionnaire (NSCQ).

#### 3.3.1 The Negative Acts Questionnaire—Revised (NAQ‐R)

The Negative Acts Questionnaire—Revised (NAQ‐R) with 32 items was first designed by Hoel and Einarsen in 2001, then revised in 2009, and with 22 questions, its psychometric properties were evaluated (Einarsen, Hoel, & Notelaers, 2009). This tool is designed to measure the amount of bullying and harassment in the workplace. The participant must imagine 6 months of work and agree or disagree based on a 5‐point Likert scale that never includes (1 point), sometimes (2 points), each month (3 points), each week (4 points) and each day (5 points). Lower scores indicate the desired status, and higher scores indicate an undesirable situation. In various studies, the validity and reliability of this questionnaire have been confirmed (Alipour, Dianat, Halvani, & Falah Zadeh, 2018; Einarsen et al., 2009; Salimi et al., 2019). In the present study, to ensure scientific validity of the NAQ‐R, content validity technique was applied. Having had internal validity, multiple‐forward translation method (Erkut, 2010) was used to translate NAQ‐R from the source language (English) into the target language (Persian). Two members translated each section; the two translations were compared and combined as the most appropriately translated and culturally approved items by the main researcher. To confirm the validity of the content, the questionnaire was given to 10 faculty members of the School of Nursing of Tehran University of Medical Sciences and based on their opinions, corrections were made. In this way, three items were removed, and two new items were added to the questionnaire, bringing the number of items to 21. The test–retest method was used to determine the tool's reliability. For this purpose, the questionnaires were given to 10 nurses who were randomly selected. Again, 2 weeks later, the questionnaire was completed by the same nurses. The correlation coefficient between the scores of test–retest scores was 0.92, which indicates the appropriate reliability of the instrument.

#### 3.3.2 Nurse self‐concept questionnaire (NSCQ)

Inspired by the Cowin Questionnaire, the NSCQ is designed to have six subscales rated based on an 8‐point Likert scale (certainly incorrect one point to completely true eight points) (Mahmoodi Shan, Rahmani, Rouhi, Vakili, & Hosseini, 2015). These subscales included public self‐concept, care, knowledge, employees’ relations, communication and leadership. This questionnaire has 36 items and higher scores indicate better the self‐concept (Shan et al., 2015). In an Iranian study, the validity of this questionnaire has been confirmed and its reliability has been established by the Cronbach's alpha method (alpha = 0.7) (Badiyepeymaye Jahromi, Keshavarzi, & Jahanbin, 2014). In the present study, to confirm the content validity, the questionnaire was given to 10 faculty members of Tehran University of Medical Sciences, and based on their opinions, corrections were made. Thus, the number of items decreased to 24 items (general self‐concept [three items], care [four items], knowledge [five items], employees’ relationships [three items], communication [four items] and leadership [five items]). The items were rated based on a five‐point Likert criterion. The test–retest method was used to determine the reliability. For this purpose, the questionnaires were completed by 10 randomly selected nurses. After 2 weeks, the questionnaires were returned to the same nurses. The correlation coefficient between the scores of the test–retest was 0.83, which indicated the appropriate reliability of the instrument.

### 3.4 Ethical consideration

After approving the research plan and obtaining a licence to conduct research from the Joint Organizational Ethics Committee of the School of Nursing and Midwifery and the Faculty of Rehabilitation of Tehran University of Medical Sciences (IR.TUMS.FNM.REC.1398.165), sampling was performed. The researchers introduced themselves to the nurses meeting the inclusion criteria and explained the purpose of the study, study procedure and confidentiality of the data and obtained their oral consent. The samples completed the self‐report questionnaires without mentioning their names.

### 3.5 Analyses

The results were analysed using SPSS version 21 and descriptive and analytical statistics. In this study, descriptive statistics were used to describe the samples, including frequency distribution tables, mean and standard deviation. Pearson correlation coefficient was used to determine the relationship between workplace bullying and professional self‐concept.

## 4 RESULTS

According to the study, about half of the nurses were female with the mean age of 32 years old. The average work experience of the samples studied in the nursing profession was nine years, and in the current department, it was four years. Most nurses surveyed (51%) were officially employed, shift working (77%) and worked in the ICU (61.5%). Most of them had a bachelor's degree (Table 1).

**TABLE 1:** Frequency distribution of nurses' demographic characteristics

Variable		*N* (%)	Mean (*SD*)
Age (years)		200	32.58 (6.38)
Nursing experience (years)		200	9.74 (5.86)
Duration of employment in the current department		200	4.59 (3.99)
Gender	Male	97 (48.5)	
	Female	103 (51.5)	
Marital status	Single	74 (37.0)	
	Married	126 (63.0)	
How to get into the profession	with awareness	36 (18.0)	
	With interest	62 (31.0)	
	Others	102 (51.0)	
Education level	Bachelor	169 (84.5)	
	Master	31 (15.5)	
Department	Emergency	41 (20.5)	
	CCU	24 (12.0)	
	ICU	123 (61.5)	
	Dialysis	12 (6.0)	
Employment status	Official	102 (51.0)	
	Treaty	31 (15.5)	
	Contractual	12 (6.0)	
	Corporate	34 (17.0)	
	Projective	21 (10.5)	
Interest in the workplace	Yes	184 (92.0)	
	No	16 (8.0)	
Forced to work in the current section	Yes	25 (12.5)	
	No	175 (87.5)	
Work shift	Morning	28 (14.0)	
	Night	18 (9.0)	
	Rotating	154 (77.0)	
Managerial post	Superviser	2 (1.0)	
	Headnurse	4 (2.0)	
	Others	194 (97.0)	

‍‍‍‍In the present study, based on the experience of daily or weekly workplace bullying, the mean score of workplace bullying experienced by the nurses in the emergency and intensive care units in terms of work‐related bullying 10.11%, person‐related bullying 4.27% and physical intimidation were 5.66%, respectively, and the total average was 6.68% (Table 2). The mean scores of the nurses’ professional self‐concept in the areas of care (17.63 *SD* 2.30), communication (17.27 *SD* 2.61), knowledge (20.64 *SD* 3.68), leadership (18.32 *SD* 4.50), employee relationships (11.22 *SD* 2.26) and nurses' general self‐concept were 11.95 *SD* 2.97, respectively. Also, the total mean score of professional self‐concept was (97.5 *SD* 15.31).

**TABLE 2:** Frequencies of NAQ‐R scale items

Variable	Never (%)	Now & then (%)	Monthly (%)	Weekly (%)	Daily (%)
Work‐related bullying					
Someone withholding information which affects your performance	34.5	52.0	4.0	3.5	6.0
Being ordered to do work below your level of competence	37.0	47.5	6.0	3.5	6.0
Having your opinions ignored	46.0	40.0	8.0	1.5	4.5
Being given tasks with unreasonable deadlines	52.5	37.0	7.5	1.5	1.5
Excessive monitoring of your work	48.0	36.5	2.5	4.0	9.0
Pressure not to claim something to which by right you are entitled (e.g. sick leave, holiday entitlement, travel expenses)	55.0	30.5	6.0	2.0	6.5
Being exposed to an unmanageable workload	27.0	33.0	11.5	10.0	18.5
Being hindered in promoting your knowledge and career	63.5	22.5	8.5	3.0	2.5
Being abused your conscience and commitment to work	57.5	31.5	3.5	3.5	4.0
Average percentage	46.77	36.72	6.38	3.61	6.5
Person‐related bullying					
Being humiliated or ridiculed in connection with your work	52.0	36.0	9.0	1.0	2.0
Having key areas of responsibility removed or replaced with more trivial or unpleasant tasks	39.0	37.0	14.5	4.0	5.5
Spreading of gossip and rumours about you	51.5	33.0	6.0	5.5	4.0
Being ignored or excluded	73.0	19.0	6.0	1.0	1.0
Having insulting or offensive remarks made about your person, attitudes or your private life	81.0	16.5	1.0	1.5	0.0
Repeated reminders of your errors or mistakes	53.5	34.5	6.5	5.5	0.0
Persistent criticism of your errors or mistakes	61.0	29.5	5.5	2.0	2.0
Practical jokes carried out by people you do not get along with	79.5	16.0	3.5	1.0	0.0
Having allegations made against you	73.0	19.5	5.0	2.5	0.0
Average percentage	62.61	26.77	6.33	2.66	1.61
Physically intimidating bullying					
Being shouted at or being the target of spontaneous anger	69.5	19.5	5.0	4.5	1.5
Intimidating behaviours such as finger‐pointing, invasion of personal space, shoving, blocking your way	71.0	19.5	4.0	4.5	1.0
Threats of violence or physical abuse or actual abuse	84.5	9.0	1.0	3.0	2.5
Average percentage	75.0	16.0	3.33	4.0	1.66

The results of this study also showed that workplace bullying is inversely related to professional self‐concept and almost all of its dimensions based on Pearson correlation coefficient (*p* = .002, *r* = −.219), that is as workplace bullying is reduced, the level of professional self‐concept increases (Table 3).

**TABLE 3:** The Relationships between Bullying and Self‐Concept

Variable	Care	Communications	Knowledge	Leadership	Employee relations	Nurses' public self‐concept	Total self‐concept
Work‐related bullying	*r*_ = −.165_	*r*_ = −.184_	*r*_ = −.245_	*r*_ = −.092_	*r*_ = −.006_	*r*_ = −.181_	*r*_ = −.178_
	*p*_ = .020_	*p*_ = .009_	*p*_ = .000_	*p*_ = .195_	*p*_ = .927_	*p*_ = .010_	*p*_ = .012_
Person‐related bullying	*r*_ = −.196_	*r*_ = −.222_	*r*_ = −.223_	*r*_ = −.111_	*r*_ = −.059_	*r*_ = −.163_	*r*_ = −.194_
	*p*_ = .005_	*p*_ = .002_	*p*_ = .001_	*p*_ = .118_	*p*_ = .406_	*p*_ = .021_	*p*_ = .006_
Physically intimidating bullying	*r*_ = −.295_	*r*_ = −.274_	*r*_ = −.287_	*r*_ = −.234_	*r*_ = −.133_	*r*_ = −.245_	*r*_ = −.297_
	*p*_ = .000_	*p*_ = .000_	*p*_ = .000_	*p*_ = .001_	*p*_ = .060_	*p*_ = .000_	*p*_ = .000_
Total bullying	*r*_ = −.212_	*r*_ = −.229_	*r*_ = −.266_	*r*_ = −.129_	*r*_ = −.047_	*r*_ = −.201_	*r*_ = −.219_
	*p*_ = .003_	*p*_ = .001_	*p*_ = .000_	*p*_ = .068_	*p*_ = .512_	*p*_ = .004_	*p*_ = .002_

## 5 DISCUSSION

The aim of this study was to determine the level of workplace bullying experienced by Iranian nurses and its relationship with professional self‐concept. The results showed that in general, the workforce bullying experienced by nurses working in emergency and intensive care units is low. In terms of work‐related bullying, on average, only about 10 per cent of nurses reported daily or weekly bullying and nearly one per cent of nurses had never experienced work‐related bullying. This rate in the person‐related and physical intimidating dimensions of bullying was about four and six per cent, respectively, which shows a very small amount. Also, 75 per cent of nurses had never experienced physical bullying. The results of this study are in line with those of a study conducted in Urmia, Iran. In that study, which was performed among 162 nurses in four hospitals in Urmia, about 70% of the nurses participating in the study had never experienced bullying and only 9% of the nurses were frequently exposed to bullying. The questionnaire used in that study was a researcher‐made questionnaire including the four dimensions of verbal, non‐verbal,, physical and performance‐related bullying (Esfahani & Shahbazi, 2014). The results of the present study are inconsistent with most studies in other countries. In a study performed in Jordan in 2018, the results showed that 43% of Jordanian nurses experienced severe bullying (Obeidat, Qan’ir, & Turaani, 2018). The results of a 2016 study in Japan revealed that 18.5% of nurses experienced daily or weekly workplace bullying (Yokoyama et al., 2016). However, in Europe and the United States, different statistics and dimensions of bullying have been reported. In Greece, approximately a third of nurses report being affected by the psychological aspects of bullying. This rate in the UK in two studies in 2014 was reported 44% and 48%, respectively (Karatza, Zyga, Tziaferi, & Prezerakos, 2017; Quine, 2001). In Russia (2018), 63% of nurses had experienced bullying in the workplace and 50% of nurses acknowledged that the phenomenon of bullying had affected their careers (Difazio et al., 2019). The prevalence of bullying in the United States and the United Kingdom was reported to be between 10% and 38%, with nurses often being bullied by directors and supervisors.

Healthcare systems seem to play an important role in the extent of bullying and its dimensions. As Obeidat et al. (2018) pointed out in his study, the lack of an organized work environment, specified tasks and scope of nurses' work, manpower, teamwork and clear policies to prevent and manage workplace bullying has led to higher rates in some Middle Eastern countries than in Western countries (Obeidat et al., 2018). In the present study, the low rate of nursing workplace bullying can be due to two reasons. One is the fact that nursing is a feminine profession in Iran and that most Iranian nurses are women, and the other is related to study environments (emergency departments and intensive care units). Nurses in the emergency and intensive care units work in these areas because they have special expertise in their field and are more content with working in these areas. In this study, 184 nurses (92% of participants) liked their workplace and 174 of the nurses (87%) admitted that they were not forced to be nurses in the current wards and that they themselves were involved in the selection of the current wards. These reasons can be of great help to nurses in adapting to working conditions and activities under low workplace bullying.

The results of this study also showed that nursing workplace bullying was inversely related to professional self‐concept and almost all its dimensions. As the amount of workplace bullying decreases, the level of professional self‐concept increases. This means that if nurses' workplace bullying is high, their professional self‐concept may decline. In the present study, self‐concept was evaluated based on the dimensions of general self‐concept, care, knowledge, employee relations and communication and leadership. The results showed that bullying and its dimensions are inversely related to professional self‐concept and almost all its dimensions. Han and Ha (2016) pointed out in her study that nurses exposed to bullying have lower self‐esteem (Han & Ha, 2016). Lee and Kim (2018) argues that workplace bullying threatens the quality of nursing services and professional self‐concept and that organizational systemic strategies should be designed and implemented to prevent this phenomenon (Lee & Kim, 2018). In studies conducted in Australia and the United States, nurses who were subjected to bullying admitted to having made an error in caring for the patient (Farrell, Bobrowski, & Bobrowski, 2006; Michelle Rowe & Sherlock, 2005). Olender (2017) showed in a study that there is an inverse relationship between nursing director care and nursing workplace bullying (Olender, 2017). In another study aimed at determining the relationship between workplace bullying and missed nursing care, the results showed that workplace bullying increase missed nursing care over two‐year period (Hogh et al., 2018). Workplace bullying can also affect teamwork in the workplace. A study in the United States found that ineffective teamwork skills could be associated with a lower incidence of workplace bullying. This study also notes that teamwork is an important tool in improving patient care quality (Logan & Michael Malone, 2018). Numerous studies have shown that workplace bullying had a negative effect on management skills and leadership of nurses and nursing managers. Hampton, Tharp‐Barrie, and Kay Rayens (2019) points out in his study that this can lead to organizational abandonment and organizational failures and is considered an important stressor in the management of nursing directors (Hampton et al., 2019). In contrast, several studies have acknowledged that directors are the most important source of bullying (Lewis & Orford, 2005; Michelle Rowe & Sherlock, 2005).

In general, it can be stated that the presence of nursing workplace bullying can negatively affect the professional self‐concept of nurses. On the other hand, the less bullying is experienced in nursing workplace, the more nurses will want to improve their careers, and thus, the quality of their work will be promoted. In contrast, when nurses experience more bullying in the workplace, they lose the desire to work and are less tolerant of patients. It is recommended that hospitals and managers use organizational and individual interventions to prevent bullying at the workplace and to always support nurses. Also, by conducting psychological tests at the time of employment, the recruitment of people who engage in bullying behaviours can be prevented.

### 5.1 Study limitations

One of the limitations of this study was the use of the self‐report method to collect data. Although it is possible to obtain more accurate information using the observation method, since the observation method has its own limitations, the self‐report method is mostly used in national and international studies.

## 6 CONCLUSION

In this study, we found that the lower is the workplace bullying level, the higher is the professional self‐concept of nurses. Although the results of this study indicate a low prevalence of bullying among Iranian nurses, due to the physical, psychological and organizational effects of bullying on nurses working in hospitals and health centres, workplace bullying experienced by nurses should not be ignored. Organizational factors that lead to bullying in nurses' work environment should also be identified. Therefore, it is recommended that more studies be conducted on the effects of workplace bullying on patient care and its dimensions, as well as the quality of care provided in hospitals and healthcare centres.

## 7 IMPLICATIONS FOR CLINICAL PRACTICE

Due to the significant relationship between workplace bullying among nurses and their professional self‐concept in the present study, nursing managers and policymakers should take effective measures to recognize the phenomenon of bullying and prevent and reduce this problem. It is suggested that further studies be conducted to investigate the causes of workplace bullying in nurses and that managers make every effort to minimize these factors.

## CONFLICT OF INTEREST

The authors have no conflict of interest to declare.

## Data Availability

The authors confirm that the data supporting the findings of this study are available within the article.
